# Primary osteoarthritis chondrocyte map of chromatin conformation reveals novel candidate effector genes

**DOI:** 10.1136/ard-2023-224945

**Published:** 2024-03-13

**Authors:** Norbert Bittner, Chenfu Shi, Danyun Zhao, James Ding, Lorraine Southam, Diane Swift, Peter Kreitmaier, Mauro Tutino, Odysseas Stergiou, Jackson T S Cheung, Georgia Katsoula, Jenny Hankinson, Jeremy Mark Wilkinson, Gisela Orozco, Eleftheria Zeggini

**Affiliations:** 1 Institute of Translational Genomics, Helmholtz Zentrum München Deutsches Forschungszentrum für Gesundheit und Umwelt, Neuherberg, Germany; 2 Centre for Genetics and Genomics Versus Arthritis, Division of Musculoskeletal and Dermatological Sciences, School of Biological Sciences, Faculty of Biology, Medicine and Health, The University of Manchester, Manchester, UK; 3 Department of Oncology and Metabolism, The University of Sheffield, Sheffield, UK; 4 Graduate School of Experimental Medicine, Technical University of Munich, München, Germany; 5 TUM School of Medicine and Health, Technical University of Munich and Klinikum Rechts der Isar, München, Germany; 6 UCL Faculty of Medical Sciences, London, UK; 7 Department of Oncology and Metabolism, University of Sheffield, Sheffield, UK; 8 NIHR Manchester Biomedical Research Centre, Manchester University NHS Foundation Trust, Manchester Academic Health Science Centre, Manchester, UK

**Keywords:** osteoarthritis, chondrocytes, osteoarthritis, knee, pharmacogenetics

## Abstract

**Objectives:**

Osteoarthritis is a complex disease with a huge public health burden. Genome-wide association studies (GWAS) have identified hundreds of osteoarthritis-associated sequence variants, but the effector genes underpinning these signals remain largely elusive. Understanding chromosome organisation in three-dimensional (3D) space is essential for identifying long-range contacts between distant genomic features (e.g., between genes and regulatory elements), in a tissue-specific manner. Here, we generate the first whole genome chromosome conformation analysis (Hi-C) map of primary osteoarthritis chondrocytes and identify novel candidate effector genes for the disease.

**Methods:**

Primary chondrocytes collected from 8 patients with knee osteoarthritis underwent Hi-C analysis to link chromosomal structure to genomic sequence. The identified loops were then combined with osteoarthritis GWAS results and epigenomic data from primary knee osteoarthritis chondrocytes to identify variants involved in gene regulation via enhancer-promoter interactions.

**Results:**

We identified 345 genetic variants residing within chromatin loop anchors that are associated with 77 osteoarthritis GWAS signals. Ten of these variants reside directly in enhancer regions of 10 newly described active enhancer-promoter loops, identified with multiomics analysis of publicly available chromatin immunoprecipitation sequencing (ChIP-seq) and assay for transposase-accessible chromatin using sequencing (ATAC-seq) data from primary knee chondrocyte cells, pointing to two new candidate effector genes *SPRY4* and *PAPPA (pregnancy-associated plasma protein A)* as well as further support for the gene *SLC44A2* known to be involved in osteoarthritis. For example, PAPPA is directly associated with the turnover of insulin-like growth factor 1 (IGF-1) proteins, and IGF-1 is an important factor in the repair of damaged chondrocytes.

**Conclusions:**

We have constructed the first Hi-C map of primary human chondrocytes and have made it available as a resource for the scientific community. By integrating 3D genomics with large-scale genetic association and epigenetic data, we identify novel candidate effector genes for osteoarthritis, which enhance our understanding of disease and can serve as putative high-value novel drug targets.

**WHAT IS ALREADY KNOWN ON THIS TOPIC**

- Osteoarthritis is a disease with a high public burden where no causal treatment exists.
- Several studies identified genetic disease risk loci, but their association to effector genes remains challenging.

**WHAT THIS STUDY ADDS**

- By analysing chromosome conformation capture data from primary chondrocyte tissue, we could identify new potential target genes for osteoarthritis-associated disease risk loci giving insights into the genetics of this complex disease.

**HOW THIS STUDY MIGHT AFFECT RESEARCH, PRACTICE OR POLICY**

- New targets for research were identified in our study and the provided dataset lay the foundation for several upcoming studies facilitating the insights into the genetics of osteoarthritis.
- These insights might serve valuable for a causal treatment of patients with osteoarthritis.

## Introduction

Osteoarthritis is a highly complex disease that poses a significant burden on both individuals and society due to its high prevalence, which can reach up to 40% in people over the age of 70 years, leading to pain, decreased mobility and associated secondary health problems.^1 2^ Current treatment approaches focus on pain relief, physiotherapy and joint replacement for end-stage disease, and causal treatments are still limited. Recent genome-wide association studies (GWAS) have made substantial progress in identifying disease risk loci, but uncovering the underlying mechanisms by which they cause disease and the translation of this knowledge into developing novel treatments remain challenging.^3^ One major hurdle in identifying the disease-causing genes associated with the identified risk loci lies in the fact that most risk variants are found in non-protein-coding regions and are enriched in tissue-specific regulatory elements, suggesting they affect the regulation of genes.^4^ Pinpointing the effector genes involved is a challenge for all complex diseases like osteoarthritis.

Gene expression in eukaryotes is known to be regulated in trans as well as in cis by enhancers that can be located over 1 Mb away from the gene they regulate.^5–8^ The current model of transcriptional activation by enhancers includes binding of several transcription factors, chromatin remodelling proteins and coactivators, to form a mediator complex, which further binds RNA polymerase II at the promoter of the regulated gene, initiating transcription.^9^ These contacts between enhancer and promoter sites can be quantified by chromatin conformation examination techniques like Hi-C, allowing us to link enhancers affected by disease-associated variants to their effector genes.^10^

In this study, we aimed to fill a gap in our understanding of osteoarthritis biology by generating the first chromosome conformation map of primary osteoarthritis patient chondrocytes. We leverage the information to glean insights into disease aetiology by linking Hi-C data with publicly available ATAC-seq,^11^ ChIP-seq^12^ and the latest large-scale osteoarthritis GWAS meta-analysis results.^3^ As gene regulation is tissue-specific,^13 14^ we focus on chondrocytes as the key cellular component of articular cartilage tissue, a major joint tissue that becomes degraded during the development of osteoarthritis.^15^

## Methods

### Chondrocyte tissue isolation

Human cartilage samples (n=9, online supplemental table 1) were collected during routine joint replacement surgery. Healthy cartilage was defined according to the International Cartilage Repair Society macroscopic grading system (grade 0–1). Cartilage samples were cut into tiny pieces and transferred into a Falcon tube, washed with phosphate-buffered saline (PBS) twice and then digested in Dulbecco’s Modified Eagle Medium with 3 mg/mL collagenase 1A (Sigma, #C9891) on a 37°C shaker overnight. Tissue suspensions were passed through a 70 µm cell strainer and single chondrocytes were collected. Cells were washed with PBS and split into aliquots. Approximately 2 million primary chondrocytes were crosslinked in 2% formaldehyde for 10 min at room temperature and the reaction was then quenched using a solution of 0.125 M glycine (Sigma, #50046) + 3% bovine serum albumin (Sigma, #A9647). Samples were then washed in PBS, snap frozen on dry ice and transferred to −80°C for long-term storage.

10.1136/ard-2023-224945.supp12 Supplementary data

### Hi-C

One million chondrocyte cells were used for library preparation using the two-restriction enzyme Arima Hi-C kit (Arima Genomics) and the KAPA HyperPrep kit (Roche #KK8504) following manufacturer’s protocols and using Illumina TruSeq dual indexes.

Library size was checked by TapeStation 4200 (Agilent) and quality control (QC) was done by QuantStudio (Life Technologies) using the NEBNext Library Quant Kit for Illumina (NEB, #E7630). Sequencing was performed on the NovaSeq6000 platform (Illumina) using NovaSeq S4 flow cells generating 150 bp paired-end reads. Samples were sequenced to an average target depth of 450 million reads per library.

### Hi-C data processing and quality control

Hi-C reads were quality filtered, trimmed and adapters were removed using fastp V.0.20.1^16^ with the default settings. Reads were processed and mapped to the GRCh38 genome with HiC-pro V.3.0.0,^17^ using default settings. For further QCing, loop and topologically associated domain (TAD) identification as well as visualisation of Hi-C contact maps, we created cooler files for a bin size of 500 kb, 50 kb, 10 kb and 5 kb corrected with the Knight-Ruiz (KR) method implemented in juicer tools with cooler V.0.8.11.^18^ To assess reproducibility between samples, we analysed cooler files with a bin size of 10 kb with HicRep.^19 20^ To identify potential outliers, we conducted principal component analysis (PCA) of the different bin-sized cooler files of the single patient samples with the PCA function implemented in FAN-C V.0.9.25^21^ and removed one sample from the downstream analysis. For downstream analysis, single patient samples were merged into a single file comprising 3.520 billion reads. Hi-C juicebox files for visualisation and further analysis were generated using the hicpro2juicebox.sh script using juicer tools V.1.22.01.^22^

### Hi-C loops identification

For identification of chromatin loop interactions, we used the KR-corrected 5 kb and 10 kb bin-sized cooler files with four different tools.

Two tools based on scale-space representation:

1. Mustache V.1.3.1,^23^ with default settings which comprise a p value threshold of 0.1.
2. SIP V.1.6.2^24^ calling juicer tools V.1.22.01,^22^ with default settings and a false discovery rate (FDR) setting of ≤0.1 comparable to the other loop algorithms used.

Two tools based on the donut approach introduced by Rao *et al* 25:

1. FAN-C V.0.9.25^21^ calculated loops were filtered with the *–rh-filter -d 5 -o 5* settings (FDR ≤0.1), merged with the *-j –remove-singlets* option and converted to the bed file format with the built in *-b* flag. Loop anchors which overlap after the merging step were removed prior to downstream analysis.
2. HiCExplorer V.3.7.2^26^ was used with standard settings except the --pValuePreselection and --pValue flag were set to 0.1 as applied for the other loop callers in this study.

For each of the tools, 5 kb and 10 kb loops were merged separately with the *hicMergeLoops* function from HiCExplorer.^27^

To assess the quality of the resulting loop datasets, we screened for CTCF binding sites based on the JASPAR 2022 database,^28^ which was extracted as a bed file with the CTCF package in R.^29^

### Topologically associated domain identification

TADs were calculated with the hicFindTADs function from HiCExplorer with cooler files of the bin size 50 kb, with a --*minDepth* setting of 3× the size of the bin and a --*maxDepth* setting of 10× the size of the bin. For multiple testing, FDR correction was set with a threshold of 0.01. Remaining optional settings were set to default values. Quality of TAD domain sites was assessed with overlaying of CTCF binding sites as described in the ‘Hi-C loops identification’ section.

### Marker gene assessment

To assess the quality of the chondrocyte tissue samples, we screened if selected marker genes for chondrocytes (online supplemental tables 5 and 6) are in an active state based on the Hi-C loops and public epigenetic data. All loop anchor regions were overlapped with enhancer and promoter elements from the latest version of the ENCODE SCREEN database for cis-regulatory elements (cCRE) (V.3).^30^ To assess which of these enhancers and promoters are in an active state, we overlaid the identified regulatory regions with public ATAC-seq^11^ (n=8) and ChIP-seq^12^ (n=3) data from primary knee chondrocytes. ATAC-seq peaks used in our analysis were identified in six out of eight patient samples from the outer region of the lateral tibial plateau which was assigned as healthy chondrocyte tissue. Promoters located in open chromatin regions were designated active when overlapping with H3K4me3 and H3K27ac peaks and enhancers with H3K4me1 and H3K27ac peaks^31^ in two out of three replicates. Only loops with active enhancer-promoter contacts were analysed. To annotate active promoters to a target gene, we searched for genes 1500 bp upstream and 500 bp downstream of the promoter region in the ENSEMBL/GENCODE database^32^ (ENSEMBL Genes 110). As ChIP-seq data were based on genomic build GRCh37, we lifted genomic coordinates with the liftover tool from the University of California, Santa Cruz (UCSC) genome browser.^33^

10.1136/ard-2023-224945.supp16 Supplementary data

10.1136/ard-2023-224945.supp17 Supplementary data

### Stratified LD score regression analysis

With stratified LD score regression (S-LDSC),^34^ we tested for heritability enrichment on all 11 osteoarthritis phenotypes reported in the largest osteoarthritis GWAS to date^3^ for all enhancers from the ENCODE SCREEN cCRE database (n=9 61 227), or enhancers overlapping the loop anchors as generated in the current study. Since GWAS summary statistics used GRCh37 reference assembly coordinates, whereas the Hi-C and enhancer regions used GRCh38, the enhancer genomic coordinates were lifted down to GRCh37 using the liftOver function from the rtracklayer package (V.1.52.1)^35^ and the appropriate UCSC chain files. The S-LDSC custom annotations, based on the enhancer definitions, were calculated using the 1000 Genomes phase III genotypes, as provided by LDSC. The S-LDSC regression for each GWAS-custom annotation pair was performed using the HapMap3 single-nucleotide polymorphism (SNP) list and the precalculated regression weights provided by LDSC. The S-LDSC regression was performed on each GWAS and the custom annotations of all enhancer regions (not filtered for overlapping loop anchors) and enhancer regions overlapping loop anchors, in addition to the baselineLD model (V.2.2), which includes 53 general functional annotations.^34^ The full results for all 22 S-LDSC regressions (11 phenotypes×2 custom annotations) are reported in the online supplemental table 7. Given the high correlation between the GWAS phenotypes tested, the enrichment p values were corrected for multiple testing by multiplying the nominal p values by the effective number of tests (Meff) (Meff=4.6565) as estimated in the original publication.

10.1136/ard-2023-224945.supp18 Supplementary data

### Linkage disequilibrium calculation

LD between the lead GWAS single nucleotide variant (SNV) and the credible set SNV, located in each loop enhancer, was calculated in plink^36^ using European individuals from the 1000 Genomes phase III data (https://www.internationalgenome.org).

### GWAS signal overlap and identification of active enhancer-promoter loops

In order to identify potential effector genes for osteoarthritis, we investigated osteoarthritis-associated variants at established GWAS loci^3^ that had a high probability (95%) of being the causal variant for each of the GWAS signals. To do this, we selected variants residing in the 95% credible sets, with a posterior probability (PP) of causality >3% (n=570). The 95% credible set of variants for each of the GWAS signals contained between 1 and 18 variants. GWAS signals containing a single variant in the 95% credible set indicate a high probability that this variant be the causal variant. Most GWAS signals contained more than one variant in the 95% credible set denoting more uncertainty surrounding which of these variants is likely to be causal. The GWAS summary statistics were generated for reference assembly GRCh37, whereas the Hi-C data were generated on GRCh38. To account for the differences in genome build, we lifted the variants in the 95% credible sets in the GWAS data from GRCh37 to GRCh38 using CrossMap^37^ (https://crossmap.sourceforge.net).

**Figure 1:**
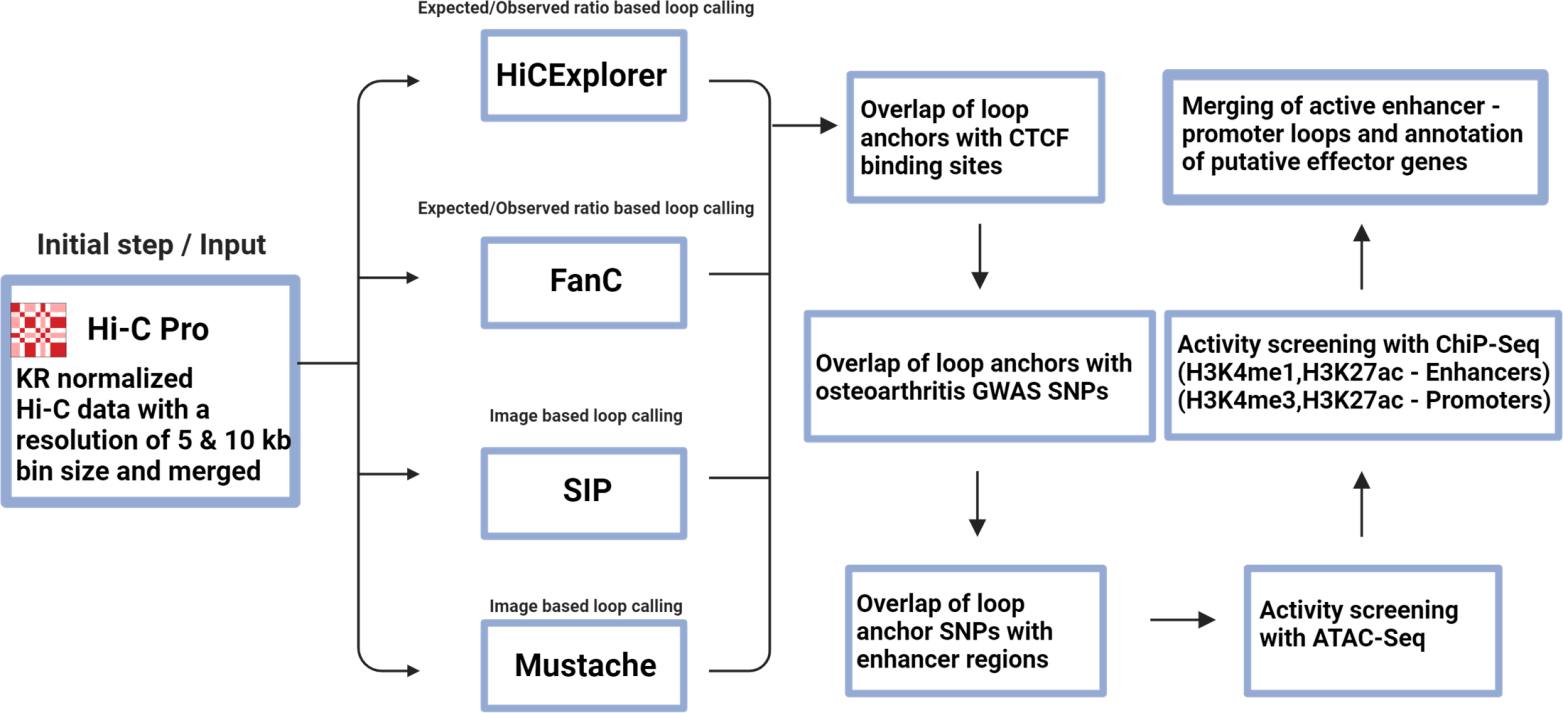
Workflow for the analysis of putative effector genes Hi-C data from primary tissue chondrocytes with a 5 kb and 10 kb bin size were Knight-Ruiz (KR) normalised, merged and analysed separately with each of the four applied algorithms. Loop anchors were overlapped with CTCF binding sites as a quality control measure (table 1). Osteoarthritis genome-wide association studies (GWAS) single-nucleotide polymorphisms (SNPs)^3^ were overlapped with loop anchors and enhancer regions from ENCODE SCREEN^30^ (V.3). Activity screening for SNP-containing enhancers was performed with overlap of public ATAC-seq^11^ and ChIP-seq data^12^. Active promoters on loop anchors contacting active SNP-enhancer regions were identified with the same epigenetic datasets. Overlapping enhancer-promoter loops were merged and putative effector genes were annotated. Figure was created with BioRender.com.

These variants were overlaid with the loop anchor positions that were identified in all four loop calling tools. We then investigated if variants residing in loop anchors overlapped with enhancer elements from the latest version of the ENCODE SCREEN cCRE database^30^ (V.3). Activity screening for these SNP harbouring enhancer elements was performed as in the marker gene assessment with ATAC-seq for open chromatin and ChIP-seq of H3K4me1 and H3K27ac signals. Contact anchor loops of these active enhancers were screened for active promoter regions to identify enhancer-promoter loops with osteoarthritis SNPs in the enhancer (figure 1). We include all genes in the vicinity of the genetic association signals as well as all identified enhancer-promoter loops resulting from our data. Overlapping loop anchors of active SNP-enhancer-promoter loops identified in one of the four algorithms were merged and used for further downstream analysis.

### Enhancer-enhancer loop analysis

For loop anchors with variants residing in an active enhancer and looping to an anchor with only active enhancer elements and no promoters, we checked if these active enhancer loop anchors loop further to an active promoter as described above.

### Loop anchors without any cCRE element

Loops without any overlap with cCRE elements in both loop anchors were screened for open chromatin and ChIP-seq signals for active enhancers to discover novel regulatory regions in chondrocytes. We further checked if these active regions show expression of enhancer-RNA in the FANTOM5 database for hg38.^38^

### Overlap with osteoarthritis molecular quantitative trait loci and osteoarthritis transcriptomics data

To establish if any of the variants in the credible set were also chondrocyte molecular quantitative trait loci (QTLs), we queried the largest available chondrocyte functional genomics datasets. We looked for expression QTLs (eQTLs), protein QTLs (pQTLs), differential protein abundance,^39^ differential expression^40^ and methylation QTLs (methQTLs).^41^ These functional datasets were generated in chondrocytes from approximately 100 individuals with knee osteoarthritis undergoing joint replacement. Each of these datasets had been generated for low-grade cartilage (macroscopically intact cartilage) and high-grade cartilage (macroscopically degraded cartilage).

### Colocalisation

If the enhancer overlapping SNV was also a molecular QTL in high-grade or low-grade cartilage, we performed colocalisation analysis to establish if the GWAS signal and the molecular QTL signal shared the same causal variant. For the GWAS summary statistics, we included all variants 1 Mb either side of the lead variant and where multiple phenotypes were associated only the lead phenotype was used, that is, the phenotype with the most significant p value.^3^ For the QTL data, we included all SNVs present in the *cis*-QTL analysis.^39 41^ We analyzed all overlapping SNVs, that is, only those present in both datasets, using coloc.fast (https://rdrr.io/github/tobyjohnson/gtx/man/coloc.fast.html) which implements the approximate Bayes factor method of Giambartolomei *et al*.^42^ The method produces five PP: PP.H0 indicating that neither trait has a genetic association in the region, PP.H1 indicating that only trait 1 has a genetic association in the region, PP.H2 indicating that only trait 2 has a genetic association in the region, PP.H3 indicating that both traits are associated, but with different causal variants and PP.H4 indicating that both traits are associated and share a single causal variant. We considered PP.H4 >0.8 as a positive indication of colocalisation.

### Transcription factor binding analysis

The variants residing in active enhancers of active enhancer-promoter loops were screened for disruption of TF binding motifs between the alleles of each variant with the web-based version of HaploReg 4.2.^43 44^

### Alpha missense

Predictions of the pathogenic effects of missense variants were performed with a web-based version of the AlphaMissense database.^45^

### Computational tools for overlapping genomic regions

Computational analysis to overlap genomic regions throughout the several steps of analysis was performed with R V.4.1^46^ with the *tidyverse* V.1.3.2^47^ and *fuzzyjoin* V.0.1.6 (https://github.com/dgrtwo/fuzzyjoin) package.

### Visualisation

Hi-C contact maps were generated from the 500 kb bin-sized KR-corrected cooler file with the hicPlotMatrix function from HiCExplorer.

Regional association plots for the GWAS signals from Boer *et al* 3 were plotted using Locus Zoom.^48^ Linkage disequilibrium was calculated UK Biobank data, European ancestry. Chromosome and position are plotted according to GRCh37/hg19. Enhancer-promoter loop regional plots were generated with pyGenomeTracks V.3.8.^49^ For visualisation of ATAC-seq and ChIP-seq signal tracks, replicate files were averaged with the *mean* function of Wiggletools^50^ and the *unionbedg* function from bedtools.^51^

Regional association plots for the GWAS signals from Boer *et al* 3 were plotted using Locus Zoom.^48^ Linkage disequilibrium was calculated UK Biobank data, European ancestry. Chromosome and position are plotted according to GRCh37/hg19. Enhancer-promoter loop regional plots were generated with pyGenomeTracks V.3.8.^49^ For visualisation of ATAC-seq and ChIP-seq signal tracks, replicate files were averaged with the *mean* function of Wiggletools^50^ and the *unionbedg* function from bedtools.^51^

Regional association plots for the GWAS signals from Boer *et al* 3 were plotted using Locus Zoom.^48^ Linkage disequilibrium was calculated UK Biobank data, European ancestry. Chromosome and position are plotted according to GRCh37/hg19. Enhancer-promoter loop regional plots were generated with pyGenomeTracks V.3.8.^49^ For visualisation of ATAC-seq and ChIP-seq signal tracks, replicate files were averaged with the *mean* function of Wiggletools^50^ and the *unionbedg* function from bedtools.^51^

### Patient and public involvement statement

There was no involvement of patients and the public in the design, conduct, reporting or dissemination plans of this research.

## Results

**Figure 2:**
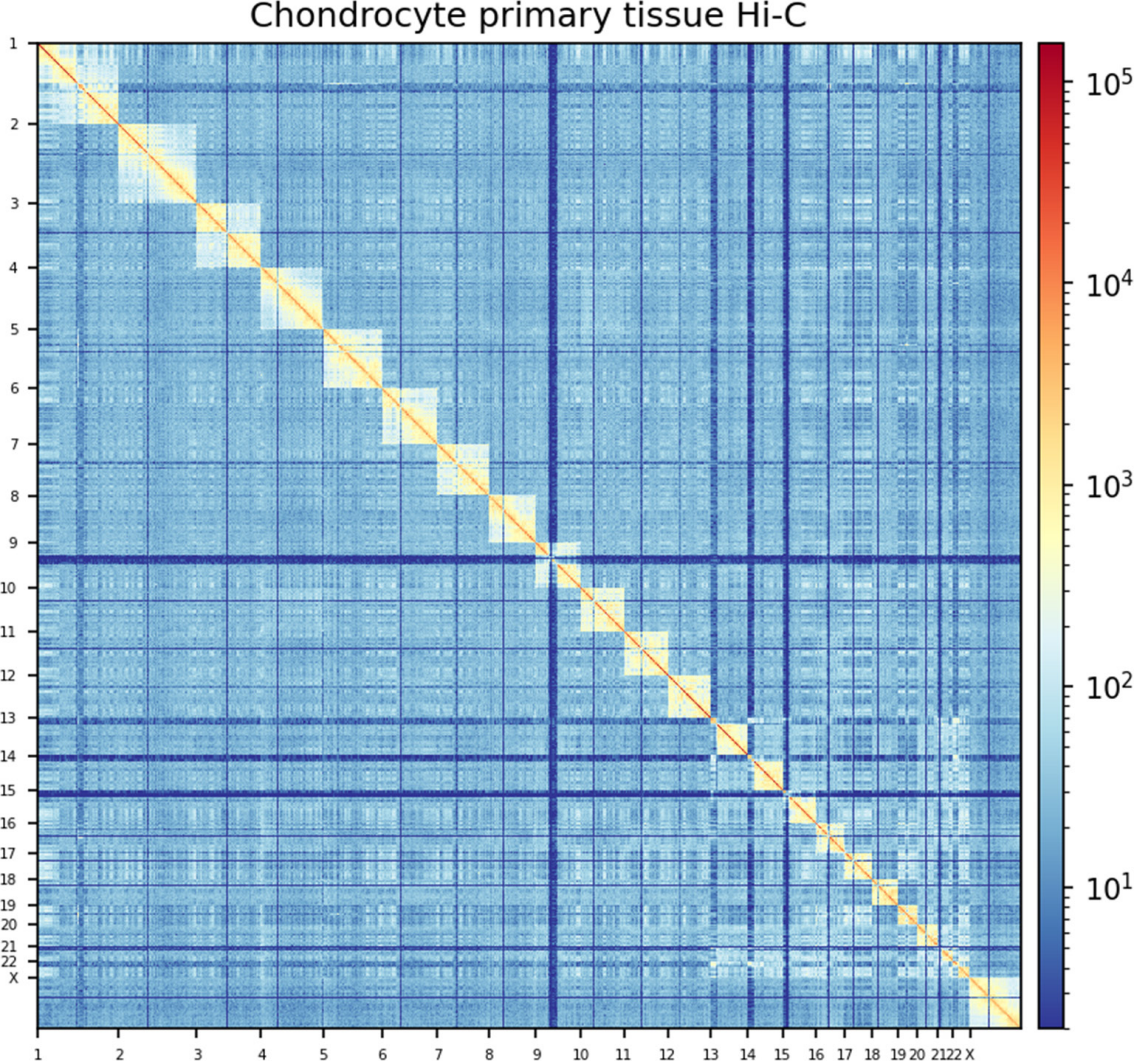
Hi-C contact matrix of primary chondrocyte tissue Hi-C contact matrix plot of all analysed chromosomes of primary chondrocyte tissue with a bin size of 500 kb. For visualisation, contact values were corrected with the Knight-Ruiz method and transformed to a log1p scale.

Analyses of the chondrocyte Hi-C data resulted in 1.75 billion unique contacts (figure 2), of which 35%–46% are *cis* long-range contacts. The between-sample reproducibility was assessed with HiCRep^19 20^ and resulted in mean stratum-adjusted correlation coefficient scores >0.75, which suggests high reproducibility between samples (online supplemental table 2). We called loops with four different loop identification algorithms and identified between 22 308–35 806 loops (table 1, online supplemental table 3). TAD identification at a resolution of 50 kb resulted in 3867 TADs (online supplemental table 4). We checked the quality of identified loops and TADs by overlaying them with CTCF binding sites as CTCF proteins are enriched within loop anchors and TAD domain boundaries.^25 52^ We observed CTCF binding sites in 75%–96% of the forward and reverse loop anchors of the different loop algorithms (table 1) and CTCF binding sites in 99.7% of the TAD domain boundaries.

10.1136/ard-2023-224945.supp13 Supplementary data

10.1136/ard-2023-224945.supp14 Supplementary data

10.1136/ard-2023-224945.supp15 Supplementary data

**Table 1:** Overview of the results of the different loop algorithms used in this study to identify chromatin loops

Loop algorithm	Loops	CTCF Fwd	CTCF Rev	EP loops	AP	AE	SNP
SIP	32 520	0.76	0.75	4605	4247	3556	10
FanC	23 844	0.96	0.96	7713	7024	12 084	10
Mustache	35 806	0.77	0.77	4587	4492	3836	6
HiCExplorer	22 308	0.79	0.80	4590	4126	3375	7

Results of the loop algorithms used in this study. Loops is the total number of loops identified. CTCF Fwd and CTCF Rev is the ratio of loop anchors with CTCF binding motifs as derived from the JASPAR 2022 database.^28^ EP loops is the number of enhancer-promoter loops with active enhancers and promoters as identified by overlap with public ATAC-seq^11^ and ChIP-seq^12^ peaks (H3K4me1, H3K4me3, H3K27ac). SNP is the number of active enhancers with a SNP from the osteoarthritis-related variants from the 95% credible sets, with a posterior probability of causality >3% from Boer *et al* 3 residing within the active enhancer region.

AE, active enhancers; AP, active promoters; SNP, single-nucleotide polymorphism.

We overlaid the identified loops with enhancers (n=961 227) and promoters (n=40 891) from the ENCODE SCREEN cCRE database^30^ (V.3) and in a further step screened if these cCRE signals are in an active state by overlapping these regulatory regions with public ATAC-seq and ChIP-seq data (H3K4me3, H3K4me1 and H3K27ac). We identified between 3375 and 12 048 active enhancer regions, between 4126 and 7024 active promoters and in total between 4587 and 7713 loops with both active enhancers and promoters (table 1).

To assess the quality of the chondrocytes, we screened for marker genes of chondrocytes within these active enhancer-promoter loops and found promoters active in suitable marker genes like *SOX9*, *COL2A1*, *GDF5* and *TPPP3*. We also checked for marker genes of prehypertrophic (*IHH*) and hypertrophic chondrocytes (*COL10A1*) but neither of these promoters was found active (online supplemental table 6).

To test whether the identified loops from chondrocytes are informative for the functional annotation of the osteoarthritis GWAS signals, we applied S-LDSC. S-LDSC, adjusted for general functional annotations,^34^ was applied to all 11 osteoarthritis phenotypes, as reported in the largest osteoarthritis GWAS meta-analysis to date.^3^ The genetic heritability enrichment was estimated for the custom annotations of all enhancers, and for the subset of enhancers overlapping loop anchors. Perhaps expectedly, after multiple testing correction, no significant enrichment was identified when using all enhancers. The enhancers overlapping loop anchors, instead, showed a strong significant enrichment in 8 out of 11 osteoarthritis phenotypes, with total hip replacement (THR) showing the largest enrichment (enrichment=8.5, adjusted p=1.2×10^−7^). Only finger, spine and thumb osteoarthritis did not show a significant enrichment, likely due to the small number of GWAS hits for these three osteoarthritis phenotypes (Nhits ≤10) (online supplemental table 7).

Given the genetic heritability enrichment identified by S-LDSC for enhancers-overlapping loop anchors, we then used the promoter-enhancer contact matrix to functionally annotate the GWAS signals from each of the 11 OA GWAS. Through integration of variants in the 95% credible set of fine-mapped signals, we identified 345 variants within 472 loop-anchor regions associated with 77 GWAS signals. The number of variants present in loops varied from 1 to 14 for each of the 77 GWAS signals, and the loops spanned a range of 30 kb to 4.0 Mb (online supplemental table 8). 14 variants are found within active enhancers of 41 loop anchors, 6–10 of these enhancers were found in each of the different loop algorithms (table 1, online supplemental table 9). Among these, 10 variants were located within 17 active enhancer-promoter loops (online supplemental table 9), and 3 variants not found in enhancer-promoter loops were present in an enhancer-enhancer loop. None of the contact loop anchors of these variants looped to an active promoter (online supplemental table 9). We additionally identified nine loop anchors which do not overlap with any cCRE element but show active epigenetic marks (online supplemental table 10). When checking for expression of enhancer RNA in the FANTOM5 database,^53^ we could not find any enhancer transcripts matching these regions. This may indicate novel, not yet identified enhancer elements specific to chondrocytes. This demonstrates that not all regulatory regions of chondrocytes are covered by cell culture models and that primary patient cell approaches are needed to complete the picture of the regulatory landscape of this cell type.

10.1136/ard-2023-224945.supp19 Supplementary data

10.1136/ard-2023-224945.supp20 Supplementary data

10.1136/ard-2023-224945.supp21 Supplementary data

### For four GWAS signals there was no clear resolution of the effector gene

On chromosome 3, lead variant rs2276749 was associated with THR and had been previously linked to the high confidence effector gene *VGLL4* (vestigial like family member 4),^3^ which is known to be involved in osteoblast differentiation^54^ and a transcriptional coactivator involved in development and disease.^55^ Two variants (rs2276749, rs6799718) of six from the 95% credible set are each located in a different active enhancer. The other variants from the credible set reside in the loop anchor showing no overlap to any other cCRE regions. The enhancer loop anchor loops to two active promoters one upstream and one downstream of the enhancers (table 2, online supplemental figures 1 and 2). One of the promoters is annotated to *VGLL4* and the lead variant, rs2276749, is a missense mutation in *VGLL4*, which is the simplest explanation for the causal mechanism for this signal, although the p.Ile32Met transition is likely benign and not directly pathogenic according to the AlphaMissense database.^56 57^

10.1136/ard-2023-224945.supp1 Supplementary data

10.1136/ard-2023-224945.supp2 Supplementary data

**Table 2:** Osteoarthritis risk-associated variants in enhancer regions of Hi-C identified enhancer-promoter loops

Lead variant (rsID)	rs2276749	rs8112559	rs143083812	rs12908498	rs10405617	rs10062749	rs1321917
Phenotype	THR	Hand OA Finger OA	THR/Hip OA	Hip OA/THR/TJR Knee-Hip OA/All OA	All OA/Knee OA	Hand OA/Thumb OA Knee OA	THR/Hip OA/TJR
Variant position	3:11601991	19:45887197	7:129203569	15:67074150	19:10642292	5:142425523	9:116562650
Credible set variants	rs2276749 rs6799718	rs8112559	rs143083812	rs1498506 rs1498507	rs10984	rs28538668 rs6861056	rs1895062
Annotated effector gene	*VGLL4*	*APOE3*	-	*SMAD3*	*ILF3* *SMARCA4*	*NR3C1*	-
Putative effector gene	-	-	-	-	SLC44A2*	*SPRY4*	*PAPPA*
Methylation and gene targets (QTLs)	-	-	-	cg09501821 eQTL (SMAD3)	cg01654627, cg17710535 eQTL (SLC44A2)	cg19514721	cg08189448
Transcription factor affected	-	IRF, Stat		Hic1, ATF3, ATF6 E2F, HEY1, Pax-4	AP-1, ATF3, E2F INSM1, Jundm2	Foxd1	AP-1, GATA, Pou2

Risk-associated phenotypes and lead variants were identified in the study by Boer *et al.* 3 Lead variants are defined as the most significantly associated SNV for each of the credible set variants, Credible set variants are variants residing in the 95% credible sets, with a posterior probability of causality >3%. Annotated effector genes were genes identified to be associated with the lead variant in the study by Boer *et al*. Putative effector genes were identified in this study. Methylation targets and its QTLs were identified in a study by Kreitmaier *et al* 41 and gene targets and their eQTLs in a study by Steinberg *et al.* 39 Transcription factors affected by the credible set variant were identified with HaploReg V.4.2.^43 44^

*Colocalisation has been previously observed in the study by Boer *et al* in Genotype-Tissue Expression data, here we add additional support for SLC44A2.

All OA, osteoarthritis at any joint site; eQTL, expression QTL; Finger OA, finger osteoarthritis; Hand OA, hand osteoarthritis; Hip OA, hip osteoarthritis; Knee-Hip OA, knee and/or hip osteoarthritis; Knee OA, knee osteoarthritis; QTL, quantitative trait loci; SNV, single nucleotide variant; THR, total hip replacement; TJR, total joint replacement.

Lead GWAS variant rs8112559 is associated with hand and also finger osteoarthritis. rs8112559 is the only variant in the credible set and is situated in an active enhancer. This signal has a single high confidence effector gene *APOE3*. In our Hi-C data, we see three different enhancer-promoter loops linking the variant to five different active promoters. None of these promoters show any association to a qTL signal. Close to the variant and the active enhancer is the gene *SYMPK* (Symplekin Scaffold Protein)*.* The protein SYMPK was reported with increased abundancy in high-grade cartilage then in low-grade cartilage^39^ but no other supportive evidence was found in any of the additional functionally relevant datasets (table 2, online supplemental figures 3 and 4). Being active in regulation of gene expression is supported by the change of two motifs of transcription factors.

10.1136/ard-2023-224945.supp3 Supplementary data

10.1136/ard-2023-224945.supp4 Supplementary data

The lead GWAS variant rs143083812 is associated with THR and also hip osteoarthritis with no high confidence effector gene assigned in the GWAS. rs143083812 is a rare missense variant situated in the *SMO* (smoothened) gene, with high PP of causality (89.6%). It also resides in an enhancer in a loop anchor connected to the promoter of *TSPAN33* (tetraspanin 33) and the alleles of rs143083812 demonstrate differential binding of four different transcription factors (table 2, online supplemental figures 5 and 6). No other evidence links this variant to *TSPAN33*. SMO is involved in Hedgehog (HH) signalling, which is important in cell patterning and differentiation, including vertebrate limbs,^58^ is associated with tissue damage repair and is activated in osteoarthritis cartilage.^59^ The genetic effect of rs143083812 and the association with osteoarthritis could be due to direct perturbation of the cholesterol-binding region in SMO via the p.Arg173Cys substitution,^56^ which activates SMO and results in increased HH activity. The mutation caused by this substitution was also rated ambiguous in the recently published AlphaMissense^56 57^ database being not directly pathogenic but still affecting the protein’s function.

10.1136/ard-2023-224945.supp5 Supplementary data

10.1136/ard-2023-224945.supp6 Supplementary data

The signal with lead variant rs12908498 on chromosome 15 is associated with hip osteoarthritis and also total joint replacement (TJR), THR, hip and/or knee osteoarthritis, and osteoarthritis at any joint site. The 95% credible set included nine variants. Two variants in the 95% credible set (rs1498506, rs1498507) are located within an active enhancer-like signature and both disrupt binding of several transcription factors (table 2). All the other variants from the credible set are located in the enhancer loop but not in any cCRE (online supplemental figures 7 and 8). The Hi-C data show one active loop from the enhancer loop anchor region pointing to the active promoters of *ICQH* and *AAGAB.* The gene identified as the putative effector gene of the GWAS variant in this region, *SMAD3* (SMAD Family Member 3) also has an active promoter but located within the loop anchor of the SNP-containing enhancer. Both rs1498506 and rs1498507 are methQTLs for the probe cg09501821 in low-grade and high-grade cartilage. cg09501821 is not annotated to a gene but it resides inside the gene region of *SMAD3* close to the transcriptional start as well as in the loop anchor with the active SNP-containing enhancer (online supplemental figures 7–9). rs1498506 and rs1498507 are also eQTLs for *SMAD3* in low-grade and high-grade cartilage. Colocalisation analysis confirmed that the GWAS signal and the eQTLs for *SMAD3* and the methQTL for cg09501821 colocalise (PP.H4 >0.8, online supplemental table 11) indicating that the signals are likely due to the same single causal variant. These results strongly support that the variants are correctly annotated to *SMAD3* as already highlighted in the study by Boer *et al*, where *SMAD3* showed the highest level of confidence of being involved in osteoarthritis.^3^ Our Hi-C data do not add another layer of evidence for this gene.

10.1136/ard-2023-224945.supp7 Supplementary data

10.1136/ard-2023-224945.supp8 Supplementary data

10.1136/ard-2023-224945.supp9 Supplementary data

10.1136/ard-2023-224945.supp22 Supplementary data

### For three GWAS signals the Hi-C data identify potential new effector genes

**Figure 3:**
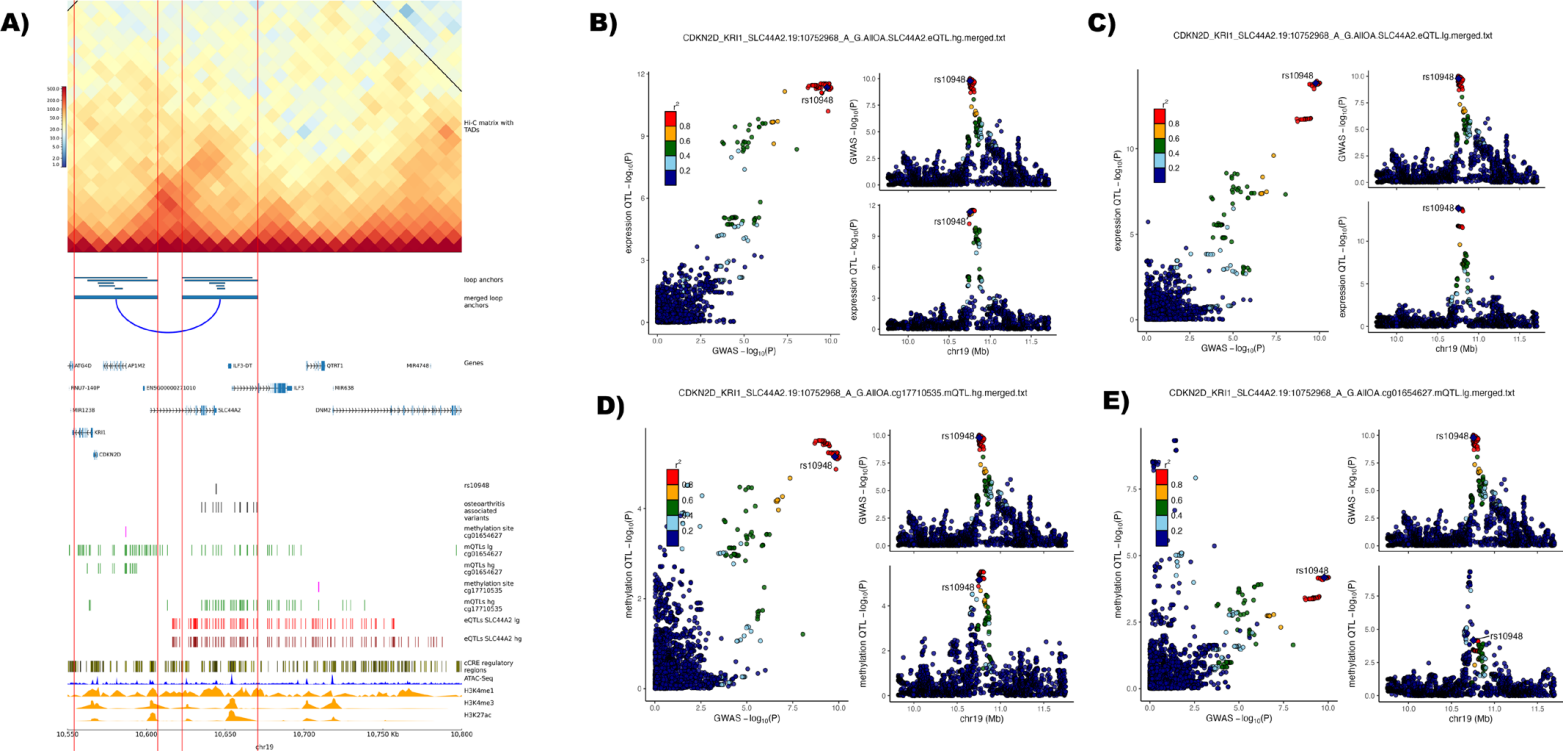
Identification of enhancer-promoter loops linked with osteoarthritis-associated lead variant rs10405617 on chromosome 19. (A) Plot of the identified enhancer-promoter loop associated with lead variant rs10405617 with the credible set variant rs10948 residing in an active cis-regulatory elements (cCRE) enhancer region. Horizontal red lines show the region of loop anchors with active promoter and enhancer regions throughout the plotting area. *Hi-C matrix with topologically associated domains (TADs)* show the log1p-transformed Hi-C contact matrix map showing the number of identified contacts between bins with a 10 kb bin size. Black lines show merged TADs calculated with a 50 kb bin size. *Loop anchors* show all identified loop anchors with the different loop calling algorithms used in this study as green bars at their respective location on the plotted chromosome region. The *merged loop anchors* show the region used for the final analysis after merging the several locally identified loop anchors. Putative identified loops are connected with a blue arc. *Genes* are the position of transcribed regions as identified in ENSEMBL genes V.110. *Osteoarthritis-associated variants* are variants from the 95% credible set of a study by Boer *et al*,^3^ with a posterior probability of >3% identified to reside in loop anchors called in this study. In addition, the position of the credible set variant residing in an enhancer region, *rs10948*, is shown in a separate track. Associated methylation QTL (methQTL) methylation sites and the respective positions of methQTLs in low-grade (lg) and high-grade (hg) degraded cartilage were identified by Kreitmaier *et al*.^41^ Positions of expression QTLs (eQTL) associated with the gene *SLC44A2* were identified in a study by Steinberg *et al.* 39 c*CRE regulatory regions* shows all cCRE as identified in V.3 from the ENCODE registry.^30^ ATAC-seq^11^ (n=8) and histone mark signal tracks for H3K4me1, H3K4me3 and H3K27ac^12^ (n=3) were averaged and merged into one track from the replicates of the public data repositories, genomic co-ordinates (GRCh38) are given below the plot. (B, C) Regional association plot with the enhancer variant rs10948 highlighted for the lead phenotype (osteoarthritis at any joint site) from Boer *et al* 3 (top right) and the eQTL for SLC44A2 in (B) hg and (C) lg cartilage^39^ (bottom right). Comparison of p values between the genome-wide association studies (GWAS) and expression QTL are depicted on the left. Variants are annotated to the enhancer variant which is highlighted in purple. Linkage disequilibrium with the lead variant is depicted according to the colours in the legend. (D, E) Regional association plot with the enhancer variant rs10948 highlighted for the lead phenotype (osteoarthritis at any joint site) from Boer *et al* 3 (top right) and the methQTL for (D) cg01654627 in hg and (E) cg01654627 in lg^41^ (bottom right). Comparison of p values between the GWAS and methQTL are depicted on the left. Variants are annotated to the enhancer variant which is highlighted in purple. Linkage disequilibrium with the lead variant is depicted according to the colours in the legend. QTL, quantitative trait loci.

Lead GWAS variant rs10405617 located on chromosome 19 is associated with osteoarthritis at any joint site and also knee osteoarthritis. There are two high confidence effector genes associated with this signal *ILF3* and *SMARCA4*. Our Hi-C data show that one of the 14 variants in the credible set (rs10948) is in an active enhancer from which the loop anchor contacts the active promoters of three genes *KRI1 (*KRI1 homolog*), CDKN2* (cyclin-dependent kinase inhibitor 2A) and *SLC44A2* (solute carrier family 44 member 2*). SLC44A2* is the closest gene to rs10948 (figure 3A, online supplemental table 9). rs10948 disrupts the binding motifs of five transcription factors (table 2). Little is known about KRI1 except being a nucleolar protein.^60^ *CDKN2* is significantly downregulated in blood from patients with osteoarthritis compared with controls and was discussed as a potential blood marker for osteoarthritis.^61^ In a study mapping osteoarthritis-related QTLs,^39^ rs1560707 was identified as an eQTL for *SLC44A2* in both high-grade and low-grade cartilage, this variant is not in the GWAS credible set. The same variant was recently shown to have an influence in the expression of *SLC44A2* due to allelic imbalance in cartilage and subchondral bone.^62^ The variant rs10948 is also a methQTL for the methylation probes cg01654627 (in low-grade cartilage) and cg17710535 (in high-grade cartilage) and an eQTL for *SLC44A2* in high-grade and low-grade cartilage. Colocalisation analysis between the GWAS and the eQTLs and methQTLs indicate that the GWAS and the eQTL and methQTL for cg17710535 are likely the same causal variant (PP.H4=0.94). The mechanistic effect of the methQTLs association with cg17710535 remains unclear as it resides outside of the loop anchor regions (figure 3B–E).

**Figure 4:**
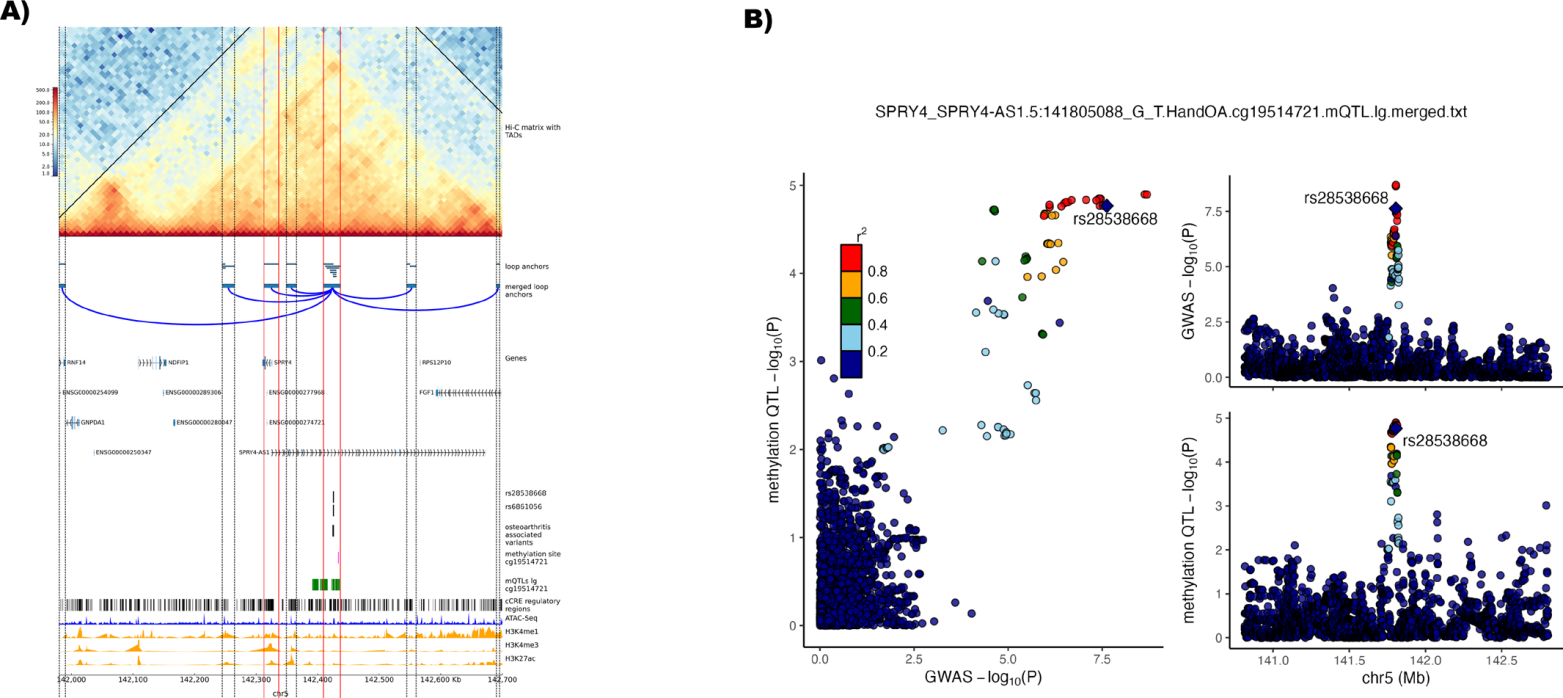
Identification of enhancer-promoter loops linked with osteoarthritis-associated lead variant rs10062749 on chromosome 5. (A) Plot of the identified enhancer-promoter loop associated with lead variant rs100062749 with the credible set variants rs28538668 and rs6861056 residing in an active cis-regulatory elements (cCRE) enhancer region. Horizontal red lines show the region of loop anchors with active promoter and enhancer regions throughout the plotting area. Horizontal dotted black lines show other identified loop anchor regions without any active enhancer-promoter region. *Hi-C matrix with topologically associated domains (TADs)* show the log1p-transformed Hi-C contact matrix map showing the number of identified contacts between bins with a 10 kb bin size. Black lines show merged TADs calculated with a 50 kb bin size. *Loop anchors* show all identified loop anchors with the different loop calling algorithms used in this study as green bars at their respective location on the plotted chromosome region. The *merged loop anchors* show the region used for the final analysis after merging the several locally identified loop anchors. Putative identified loops are connected with a blue arc. *Genes* are the position of transcribed regions as identified in ENSEMBL genes V.110. *Osteoarthritis-associated variants* are variants from the 95% credible set of a study by Boer *et al*,^3^ with a posterior probability of >3% identified to reside in loop anchors called in this study. In addition, the position of the credible set variants residing in an enhancer region rs28538668 and rs6861056 are shown in a separate track. Associated methylation QTL (methQTL) methylation sites and the respective positions of methQTLs in low-grade (lg) degraded cartilage were identified by Kreitmaier *et al.* 41 c*CRE regulatory regions* shows all cCRE as identified in V.3 from the ENCODE registry.^30^ ATAC-seq^11^ (n=8) and histone mark signal tracks for H3K4me1, H3K4me3 and H3K27ac^12^ (n=3) were averaged and merged into one track from the replicates of the public data repositories, genomic co-ordinates (GRCh38) are given below the plot. (B) Regional association plot with the enhancer variant rs28538668 highlighted for the lead phenotype (hand osteoarthritis) from Boer *et al* 3 (top right) and the methQTL for cg19514721 in lg^41^ (bottom right). Comparison of p values between the genome-wide association studies (GWAS) and methQTL are depicted on the left. Variants are annotated to the enhancer variant which is highlighted in purple. Linkage disequilibrium with the lead variant is depicted according to the colours in the legend. QTL, quantitative trait loci.

Lead variant rs10062749 at chromosome 5 is associated with hand and also thumb and knee osteoarthritis and consists of four credible set variants of which two (rs28538668, rs6861056) are in an active enhancer. The other two variants can be found in the loop anchor but do not overlap with any identified cCRE. This GWAS signal had a single high-confidence effector gene associated with it*—NR3C1* which encodes the glucocorticoid receptor. Endogenous glucocorticoids play a role in the disease progression of osteoarthritis.^63^ Our Hi-C data reveal a single loop connecting the active promoters of *SPRY4* (sprouty RTK signalling antagonist 4) and/or the promoter of the *SPRY4-antisense RNA 1* (figure 4A, online supplemental table 9 and online supplemental figure 10), with active enhancers with the credible set variants identified. SPRY4 is an inhibitor of mitogen-activated protein kinase and it has been shown that high levels of *SPRY4* expression in human cartilage prevented hypertrophy of chondrocytes.^64^ In addition, rs28538668 is a methQTL associated with cg19514721 which is located in the promoter loop anchor around 8 kb upstream of rs28538668 enhancer site and the alleles of rs28538668 are predicted to disrupt the motif for the binding of transcription factor Foxd1 (figure 4B, table 2). Colocalisation analysis showed that the GWAS signal and the methQTL signal for cg19514721 in low-grade cartilage colocalise (PP.H4 >0.8, online supplemental table 11).

**Figure 5:**
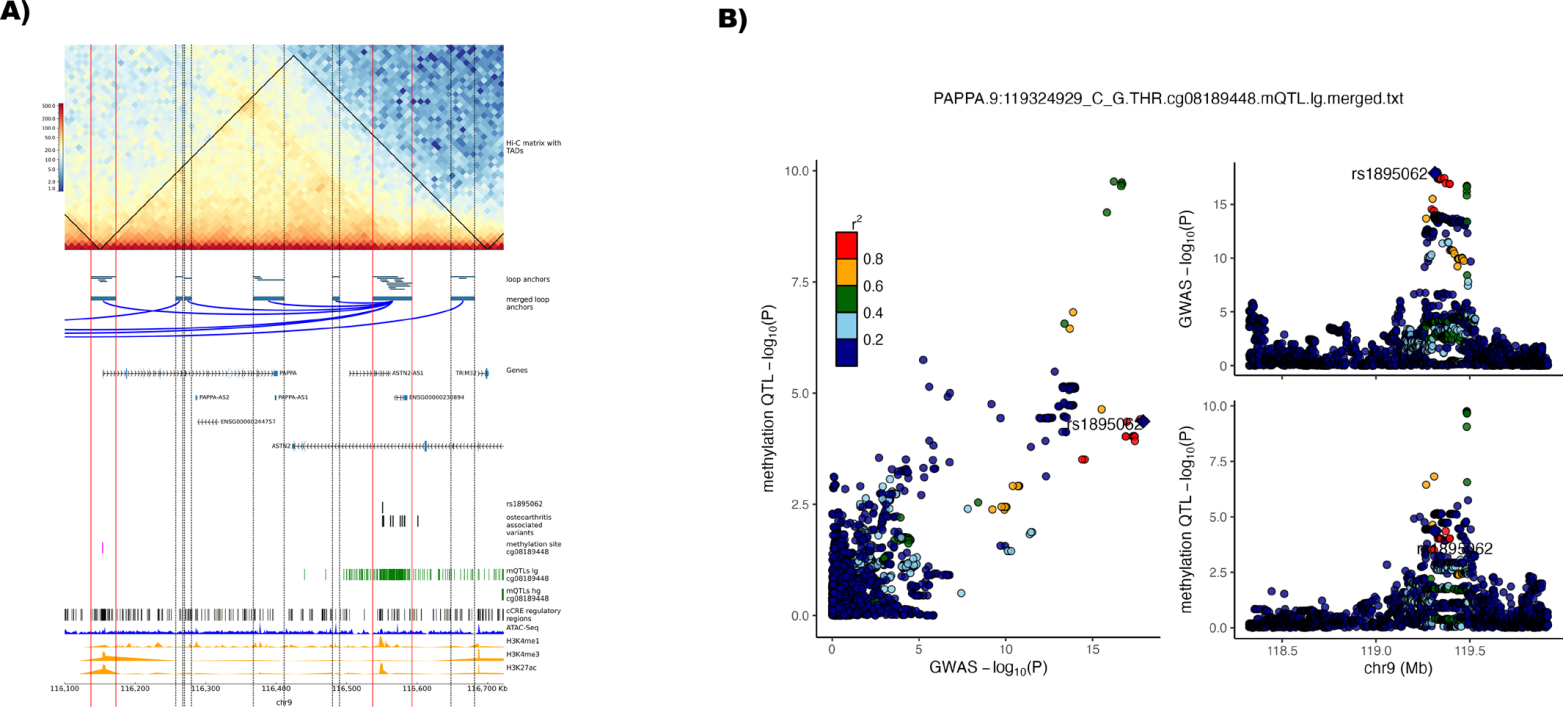
Identification of enhancer-promoter loops linked with osteoarthritis associated lead variant rs1321917 on chromosome 9. (A) Plot of the identified enhancer-promoter loop associated with lead variant rs1321917 with the credible set variant rs1895062 residing in an active cis regulatory elements (cCRE) enhancer region. Horizontal red lines show the region of loop anchors with active promoter and enhancer regions throughout the plotting area. Horizontal dotted black lines show other identified loop anchor regions without any active enhancer/promoter region. *Hi-C matrix with topologically associated domains (TADs)* show the log1p-transformed Hi-C contact matrix map showing the number of identified contacts between bins with a 10 kb bin size. Black lines show merged TADs calculated with a 50 kb bin size. *Loop anchors* show all identified loop anchors with the different loop calling algorithms used in this study as green bars at their respective location on the plotted chromosome region. The *merged loop anchors* show the region used for the final analysis after merging the several locally identified loop anchors. Putative identified loops are connected with a blue arc. *Genes* are the position of transcribed regions as identified in ENSEMBL genes V.110. *Osteoarthritis-associated variants* are variants from the 95% credible set of a study by Boer *et al*,^3^ with a posterior probability of >3% identified to reside in loop anchors called in this study. In addition, the position of the credible set variant residing in an enhancer region, rs1895062, is shown in a separate track. Associated methylation QTL (mQTL) methylation sites and the respective positions of mQTLs in low-grade (lg) and high-grade (hg) degraded cartilage were identified by Kreitmaier *et al.* 41 c*CRE regulatory regions* shows all cCRE as identified in V.3 from the ENCODE registry.^30^ ATAC-seq^11^ (n=8) and histone mark signal tracks for H3K4me1, H3K4me3 and H3K27ac^12^ (n=3) were averaged and merged into one track from the replicates of the public data repositories, genomic co-ordinates (GRCh38) are given below the plot. (B) Regional association plot with the enhancer variant rs1895062 highlighted for the lead phenotype (total hip replacement) from Boer *et al* 3 (top right) and the mQTL for cg08189448 in lg^41^ (bottom right). Comparison of p values between the GWAS and mQTL are depicted on the left. Variants are annotated to the enhancer variant which is highlighted in purple. Linkage disequilibrium with the lead variant is depicted according to the colours in the legend. QTL, quantitative trait loci.

Lead variant rs1321917 on chromosome 9 is associated with THR, hip osteoarthritis and TJR.^3^ There were no high confidence effector genes identified for this signal in the GWAS publication. Four variants from the 95% credible set are located within one loop anchor and one of the four variants (rs1895062) is found within an active enhancer-like signature disrupting motifs of three different transcription factors (table 2). The loop links to the promoter of *PAPPA* (pregnancy-associated plasma protein A) (figure 5, online supplemental table 9 and online supplemental figure 11). In addition, rs1895062 is a methQTL in low-grade cartilage for cg08189448, a methylation site located in the promoter region of *PAPPA* (table 2). Furthermore, the osteoarthritis risk allele is associated with hypomethylation of cg08189448.^41^ Colocalisation analysis revealed that the GWAS signal and the methQTL signal for cg08189448 are likely driven by the same single causal variant (PP.H4 >0.8, online supplemental table 11). PAPPA was first identified as a plasma protein from the placenta but was later shown to be involved in several biological processes including regulation of insulin-like growth factor (IGF) bioavailability in many tissues.^65^ In a gene expression study conducted in primary cartilage samples from patients undergoing joint replacement in hip and knee joints, it was shown that *PAPPA* was upregulated in osteoarthritis affected cartilage, specifically in hip osteoarthritis. This is in concordance with the GWAS THR, hip and TJR association for this signal.^3 66^ PAPPA cleaves the IGF-1 transporter proteins IGFBP4 and IGFBP5 and releases IGF-1 to its receptor.^67^ Hypomethylation is generally related to increased activity of gene expression and high levels of PAPPA result in increased cleavage of IGFBP5. A study conducted in dogs treated with protease inhibitors showed that increased levels of intact IGFBP5 result in an improvement in joint architecture during development of osteoarthritis,^68^ indicating a negative role of increased levels of PAPPA. In an in vitro model in which human chondrocytes cells were cultured and exposed to fibronectin fragments (FN-f) in order to mimic osteoarthritis, the expression of *PAPPA* was shown to be increased after FN-f exposure.^69^ In PAPPA-null mice, there was marked increase rate of bone formation^70^ and homozygous knockouts for *PAPPA* were 40% smaller than wild-type littermates at birth.

10.1136/ard-2023-224945.supp11 Supplementary data

## Discussion

In this work, we have generated the first chromosome conformation map of primary osteoarthritis patient chondrocytes.

We demonstrate that integrating tissue-specific Hi-C data with genome-wide association findings and epigenetic data adds another layer of evidence and provides additional insights into the regulatory landscape of osteoarthritis. Several studies have provided insights into the aetiology of complex diseases such as type 2 diabetes,^71^ multiple sclerosis^72^ and different types of cancer^73–75^ by adding conformation capture data to identify causal genes for GWAS variants associated with these diseases. For osteoarthritis, a recent study identified a potential novel anti-inflammatory gene by integrating Hi-C data from cultured chondrocyte cells with CUT&RUN and RNA-sequencing data from fibronectin-treated cells.^76^ Examining chromosomal conformation in primary tissue offers advantages, as it accurately captures the in vivo spatial organisation of chromosomes, closely resembling the actual regulatory conditions in the disease-relevant cell types. To date, no Hi-C data of ex vivo primary chondrocyte cells have been available. With this work, we have filled this gap, and have made the first Hi-C map of primary osteoarthritis chondrocytes available to the wider scientific community (DSR798SDK at the Musculoskeletal Knowledge portal and GSE260760 at the NCBI GEO repository). By integrating these data with GWAS summary statistics, we could identify several as yet unknown targets of osteoarthritis-associated variants found in enhancer-promoter loops. For example, we have identified *PAPPA* as a high-confidence effector gene. A study using Hi-C analysis in primary human haematopoietic cells also linked this lead variant to the promoter of *PAPPA.* 77 *PAPPA* is directly involved in the cleavage of two IGF-1 transport proteins^67^ and activation of *PAPPA* results in an increase of bioactive IGF-1.^78^ IGF-1 is a crucial factor in the repair of damaged chondrocytes and may be a potential component for the treatment of osteoarthritis.^79^

From a technical perspective, we have shown that there remains a limitation in the computational capabilities of loop chromatin detection in Hi-C datasets reflected by different results in quantitative (table 1) as well as qualitative aspects with limited overlap of active enhancer-promoter loops between the different loop detection algorithms (online supplemental table 9). Still, quality metrics of all loop callers except for a noticeable high rate of enrichment of CTCF binding sites in the FanC tool are within an established range as reported in the seminal publication by Rao *et al*.^25^ We have therefore chosen to add together the results of the different loop callers and integrate these before continuing with downstream analysis. Future improvements in loop detection algorithms will increase the number of reliable loops available for integrating GWAS data. Furthermore, a comprehensive regulatory map of chondrocytes is currently missing. Such a tissue-specific enhancer map will be able to contribute to additional insights into the genetic basis of osteoarthritis in combination with the Hi-C data presented here.

In conclusion, our study has provided insights into the complex nature of osteoarthritis and its genetic underpinning. By employing chromosome conformation capture analysis (Hi-C) in primary osteoarthritis chondrocytes, we have successfully identified genes that likely play a role in the development and progression of the disease. Through the integration of three-dimensional (3D) genomics with genetic association and primary patient chondrocyte epigenetic data, we have pinpointed high-confidence osteoarthritis effector genes, which hold potential as novel therapeutic targets. Generation of the first Hi-C map of primary human chondrocytes contributes to our understanding of chromosome organisation in 3D space and sheds light into the intricate interplay between genomic structure and disease susceptibility in osteoarthritis. By openly sharing this resource with the scientific community, we aim to encourage further studies leveraging these data and hope to further advance our understanding of osteoarthritis aetiopathogenesis.

10.1136/ard-2023-224945.supp10 Supplementary data

## Data Availability

Data are available in a public, open access repository. Data used for this study are made available on the Musculoskeletal Knowledge portal under the accession DSR798SDK and at the NCBI GEO portal under accession number GSE260760.
