# Supplementary material for: Primary osteoarthritis chondrocyte map of chromatin conformation reveals novel candidate effector genes
Source: Ann Rheum Dis. 2024 Mar 13;83(8):1048–59. doi: 10.1136/ard-2023-224945 (PMC11287644; doi:10.1136/ard-2023-224945)
Supplement: Supplementary data [file ard-2023-224945supp006.pdf]

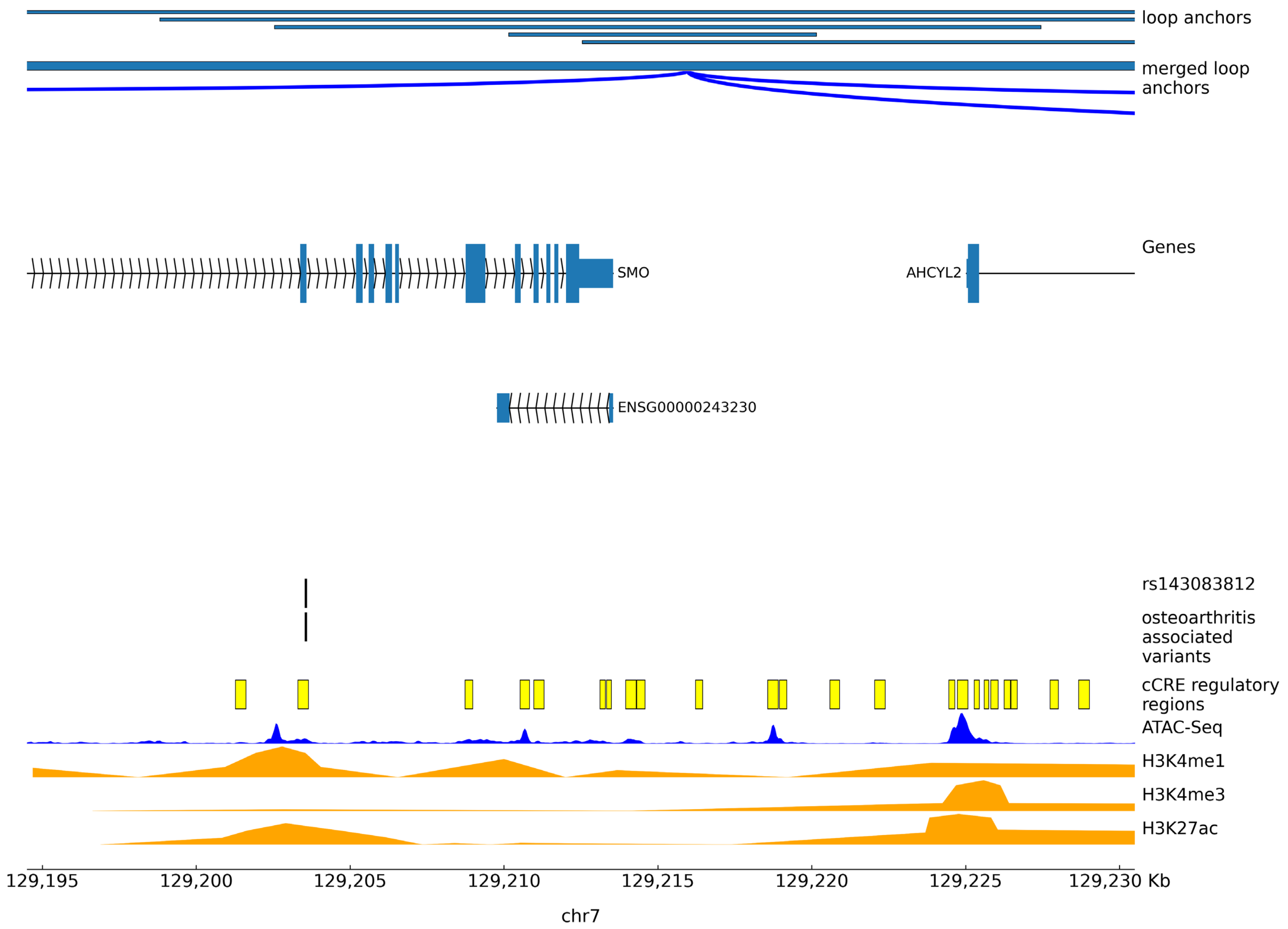

Supplemental Figure 6 **Identification of enhancers linked with osteoarthritis lead variant rs143083812 on chromosome 7**
Zoomed in plot of the enhancer residing lead variant rs143083812. Putative identified loop regions are connected with a blue arc. Loop anchors show all identified loop anchors with the different loop calling algorithms used in this study as green bars at their respective location on the plotted chromosome region. The merged loop anchors show the region used for the final analysis after merging the several locally identified loop anchors. *Genes* are the position of transcribed variants in the plotted region as identified in ENSEMBL genes version 110. *Osteoarthritis associated variants* are variants from the 95% credible set of a study by Boer, Hatzikotoulas, Southam et al.[3] with a posterior probability of > 3% identified to reside in loop anchors called in this study. In addition, the position of the credible set variants residing in an enhancer region, rs143083812 is shown in a separate track. c*CRE regulatory regions* shows all cis regulatory elements (cCRE) as identified in version3 from the ENCODE registry[30]. ATAC Seq[11] (n = 8) and histone mark signal tracks for H3K4me1, H3K4me3 and H3K27ac[12] (n = 3) were averaged and merged into one track from the replicates of the public data repositories, Genomic co-ordinates (GRCh38) are given below the plot.
