# Supplementary material for: Primary osteoarthritis chondrocyte map of chromatin conformation reveals novel candidate effector genes
Source: Ann Rheum Dis. 2024 Mar 13;83(8):1048–59. doi: 10.1136/ard-2023-224945 (PMC11287644; doi:10.1136/ard-2023-224945)
Supplement: Supplementary data [file ard-2023-224945supp007.pdf]

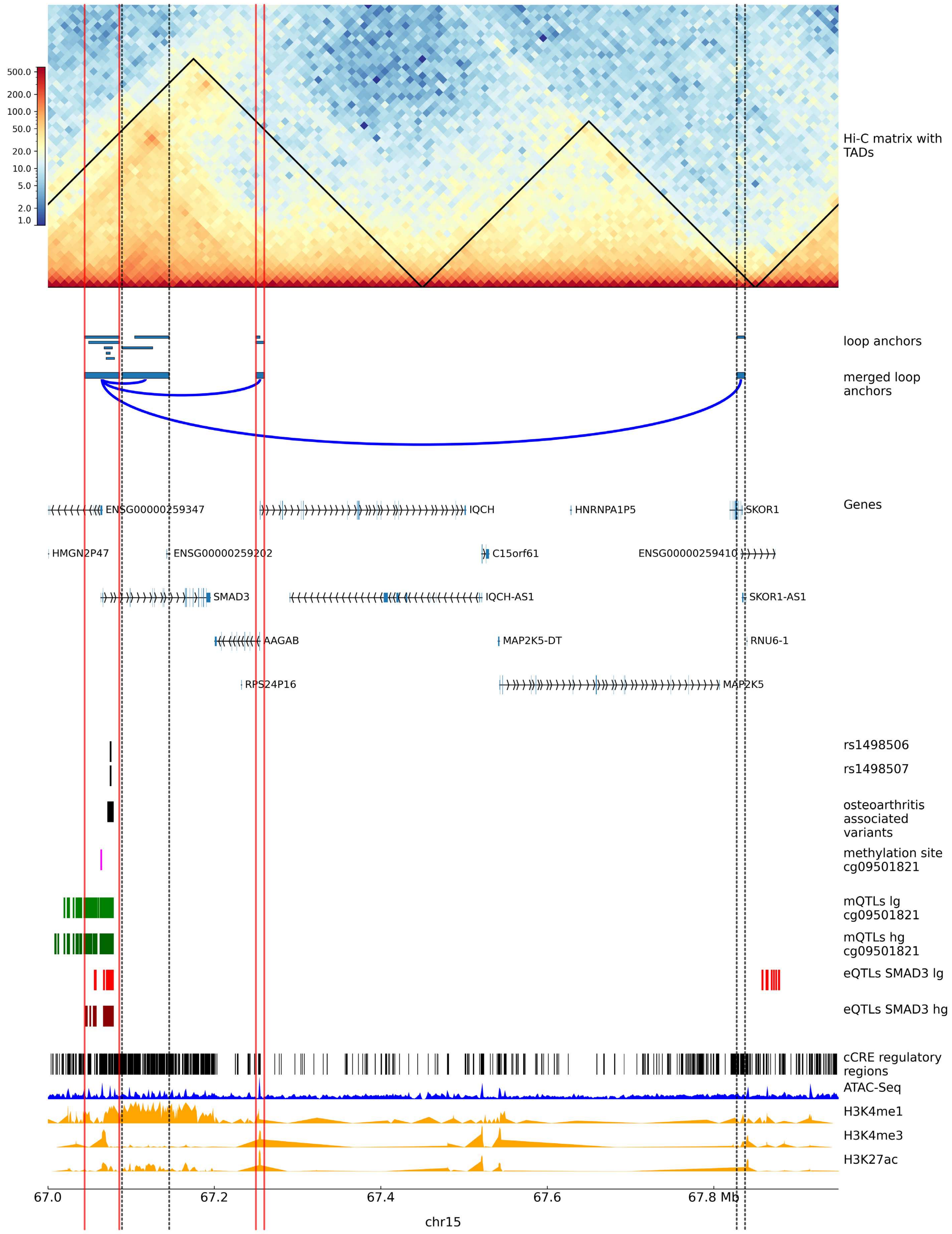

Supplemental Figure 7 **Identification of enhancer – promoter loops linked with osteoarthritis lead variant rs12908498 on chromosome 15**
Plot of the identified enhancer – promoter loop associated to lead variant rs12908498. Horizontal red lines show the region of loop anchors with active promoter and enhancer regions throughout the plotting area. Horizontal dotted black lines show other identified loop anchor regions without any active enhancer/promoter region. *Hi-C matrix with TADs* shows the identified loop region as a log1p-transformed Hi-C contact matrix map showing the number of identified contacts between bins with a 10 kb bin size. Black lines shows merged topologically associated domains (TADs) calculated 50 kb bin sizeLoop anchors show all identified loop anchors with the different loop calling algorithms used in this study as green bars at their respective location on the plotted chromosome region. The merged loop anchors show the region used for the final analysis after merging the several locally identified loop anchors. Putative identified loop regions are connected with a blue arc. *Genes* are the position of transcribed regions as identified in ENSEMBL genes version 110. Genes mentioned in the main text are marked with a red box. *Osteoarthritis associated variants* are variants from the 95% credible set of a study by Boer, Hatzikotoulas, Southam et al.[3] with a posterior probability of > 3% identified to reside in loop anchors called in this study. In addition, the position of the credible set variants residing in an enhancer region, rs1498506 and rs1498507 are shown in a separate track. Associated methylation QTL (mQTL) methylation sites and the respective positions of mQTLs in low grade (lg) and high grade (hg) degraded cartilage were identified by Kreitmaier et al.[41]. Positions of expression QTLs (eQTL) associated with the gene *SMAD3* were identified in a study by Steinberg et al.[39]. c*CRE regulatory regions* shows all cis regulatory elements (cCRE) as identified in version3 from the ENCODE registry[30]. ATAC Seq[11] (n = 8) and histone mark signal tracks for H3K4me1, H3K4me3 and H3K27ac[12] (n = 3) were averaged and merged into one track from the replicates of the public data repositories, Genomic co-ordinates (GRCh38) are given below the plot.
