# Supplementary figures and images for: Primary osteoarthritis chondrocyte map of chromatin conformation reveals novel candidate effector genes
Source: Ann Rheum Dis. 2024 Mar 13;83(8):1048–59. doi: 10.1136/ard-2023-224945 (PMC11287644; doi:10.1136/ard-2023-224945)

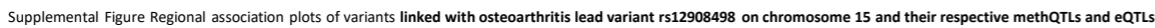

Bittner N, *et al.* *Ann Rheum Dis* 2024; 83:1048–1059. doi: 10.1136/ard-2023-224945

Supplement: Supplementary data [file ard-2023-224945supp009.pdf]
